# Consumption of Ultra-Processed Foods Increases the Likelihood of Having Obesity in Korean Women

**DOI:** 10.3390/nu13020698

**Published:** 2021-02-22

**Authors:** Hyuni Sung, Ji Min Park, Se Uk Oh, Kyungho Ha, Hyojee Joung

**Affiliations:** 1 Department of Public Health, Graduate School of Public Health, Seoul National University, Seoul 08826, Korea; hyunis@snu.ac.kr (H.S.); jmin0211@snu.ac.kr (J.M.P.); ohseuk93@snu.ac.kr (S.U.O.); 2 Department of Food Science and Nutrition, Jeju National University, Jeju 63243, Korea; kyungho.ha@jejunu.ac.kr; 3 Institute of Health and Environment, Seoul National University, Seoul 08826, Korea

**Keywords:** ultra-processed foods, obesity, abdominal obesity, Korea National Health and Nutrition Examination Survey

## Abstract

This study aimed to determine the association between consumption of ultra-processed foods and obesity among Korean adults. We used the data of 7364 participants (men 3219, women 4145) aged 19–64 years from the Korea National Health and Nutrition Examination Survey (KNHANES), 2016–2018. Food items were classified using the NOVA food classification system, depending on the extent and purpose of food processing: (1) unprocessed or minimally processed foods, (2) processed culinary ingredients, (3) processed foods, and (4) ultra-processed foods. Consumption of ultra-processed foods accounted for 26.8% of the total energy intake. After adjusting for potential confounders including sociodemographic and lifestyle characteristics, subjects with the highest consumption of ultra-processed foods (fourth quartile of % energy intake from ultra-processed foods) had 0.61 kg/m^2^ higher body mass index (BMI; 95% confidence interval [CI] 0.23–0.99, *p*-trend 0.0047), 1.34 cm higher waist circumference (WC; 95% CI 0.35–2.34, *p*-trend 0.0146), 51% higher odds of being obese (BMI > 25 kg/m^2^; odds ratio [OR] 1.51, 95% CI 1.14–1.99, *p*-trend 0.0037), and 64% higher odds of abdominal obesity (men: WC ≥ 90 cm, women: WC ≥ 85 cm; OR 1.64, 95% CI 1.24–2.16, *p*-trend 0.0004) than those with the lowest consumption (first quartile) among women. However, no association was found in men. These findings provide evidence that high consumption of ultra-processed foods is positively associated with obesity in Korean women. Further studies with a large-scale cohort or intervention trial are needed to identify the mechanism of associations between consumption of ultra-processed foods and health-related outcomes including obesity in Korea.

## 1. Introduction

The World Health Organization (WHO) has reported that the worldwide prevalence of obesity has nearly tripled since 1975, and 13% of adults aged ≥18 years were obese in 2016 [1]. At the same time, global diets have shifted remarkably from traditional diets based on freshly prepared meals to modern diets composed of high amounts of packaged and processed foods [2]. Some ecological studies have shown that consumption of ultra-processed foods has increased continuously, and this shift in modern diets has coincided with an increasing prevalence of obesity [3]. Globally, sales of ultra-processed products increased by 43.7% from 2000 to 2013 [4]. Ultra-processed foods account for almost 50–60% of calories consumed in Canada [5], the USA [6], and the UK [7], and approximately 20–30% in Brazil [8] and Chile [9]. Consumption of ultra-processed foods is currently higher in high-income countries, but it has been steadily increasing in middle-income countries [10].

The concept of ultra-processed foods is one of the groups in the NOVA system, which was first introduced by Monteiro and colleagues at the University of São Paulo. The NOVA system classifies all food items into four groups according to the nature, extent, and purpose of industrial food processing: Group 1—unprocessed/minimally processed foods; Group 2—processed culinary ingredients; Group 3—processed foods; Group 4—ultra-processed food [11].

Ultra-processed foods are formulations of ingredients, mostly of exclusive industrial use, typically made by a series of industrial techniques and processes [12,13]. These food processing techniques and ingredients are usually used to make the food convenient, highly profitable, palatable, and more appealing [11]. Examples of ultra-processed foods are carbonated drinks, packaged snacks, confectioneries, mass-produced packaged breads, ready-to-eat products, reconstituted meats, and instant soups and noodles [11,12].

Ultra-processed foods often have a higher energy density, added sugar, sodium, and fat, and lower dietary fiber and micronutrients [14]. Previous studies reported that higher consumption of ultra-processed foods is associated with adverse health outcomes such as metabolic syndrome [15], cardiovascular disease [16], depression [17], cancer [18], and mortality [19,20]. Particularly, there is a growing body of evidence supporting a positive association between ultra-processed foods and obesity. Several nationally representative cross-sectional studies have shown a significant association between consumption of ultra-processed foods and higher body mass index (BMI) or waist circumference (WC) and the prevalence of obesity in Australia [21], Brazil [22], Canada [23], the USA [24], and the UK [25]. In addition, in prospective cohort studies conducted in France [26], Spain [27], and the UK [28], participants with higher consumption of ultra-processed foods presented a higher BMI or higher risk of being overweight or obese than those with lower consumption. Recently, a randomized controlled study conducted in the USA showed that higher consumption of ultra-processed foods caused increased energy intake and body weight gain [29].

The traditional Korean diet is generally composed of rice and vegetables; however, it has been diversified by the increasing availability of processed foods and the growth of the fast food market [30,31]. Particularly, consumption of sugar-sweetened beverages (from 32 to 82 kcal/d) and fast food (from 14 to 19 kcal/d) has rapidly increased from 1998 to 2009 [30]. Furthermore, from 2009 to 2015, the prevalence of obesity increased from 29.7% to 32.4% and that of abdominal obesity increased from 18.4% to 20.8% in Korean adults [32]. In this regard, some studies have been performed to determine the association between consumption of processed foods and obesity in Korea; however, these studies were limited to consumption of individual processed foods such as sugar-sweetened beverages [33], fast food, and instant noodles [34,35].

To the best of our knowledge, no studies have evaluated the relationship between the consumption of ultra-processed foods and obesity using the NOVA classification in Korea. Therefore, this study was conducted to evaluate the consumption of ultra-processed foods and its association with obesity among Korean adults, using nationally representative data from the Korea National Health and Nutrition Examination Survey (KNHANES).

## 2. Materials and Methods

### 2.1. Data Source and Subjects

We used the 7th KNHANES data from the period of 2016–2018. The KNHANES is a representative cross-sectional survey that assesses the health and nutritional status of the Korean population and is composed of three sections: the Health Interview Survey, the Health Examination Survey, and the Nutrition Survey. This survey used a clustered, multistage, stratified, and rolling sampling design to represent non-institutionalized civilian Koreans. Detailed information about the KNHANES has been provided elsewhere [36]. The data collection protocol was approved by the Institutional Review Board of the Korea Centers for Disease Control and Prevention, and written informed consent was obtained from all participants. This study did not require additional Institutional Review Board approval because the KNHANES dataset is a publicly available de-identified dataset.

Among 14,433 subjects aged 19–64 years with available 24-h dietary recall data who participated in the 7th KNHANES (2016–2018), we sequentially excluded subjects who were pregnant or breastfeeding (*n* = 196); who had missing information on height, weight, and WC (*n* = 748); who reported implausible energy intake (<500 or >5000 kcal/day) or answered that they were on a diet (*n* = 5166); who did not have enough information on the ingredients of reported food and dishes to classify them into the NOVA food groups (*n* = 959). Finally, a total of 7364 subjects (3219 men and 4145 women) were included for analysis.

### 2.2. Obesity Indicator

In the KNHANES, participants’ height, weight, and WC were measured by trained health technicians using a standardized protocol [36,37]. BMI was calculated by dividing body weight (kg) by the square of height (m^2^). Obesity (≥25 kg/m^2^) and abdominal obesity (WC ≥ 90 cm in men and ≥85 cm in women) were defined according to the criteria of the WHO for the Asia-Pacific region [38] and the Korean Society for the Study of Obesity [39], respectively.

### 2.3. Dietary Assessment

The nutrition survey was conducted in the subject’s homes a week after the health interview and health examination survey. The surveys were performed on either weekdays or weekends during all four seasons. Dietary data were collected using the 24-h dietary recall method, administered through face-to-face interviews by trained interviewers.

Information on the names of foods and dishes, amount of food consumed, recipes, and brand names of processed foods was collected. Daily energy and nutrient intakes of subjects were calculated from the Korean Food Composition Tables published by the Rural Development Administration [40].

All recorded food items or underlying ingredients of mixed dishes were classified into one of four groups using the NOVA food classification system, proposed by Monteiro et al. [11,12]: Group 1—unprocessed/minimally processed foods; Group 2—processed culinary ingredients; Group 3—processed foods; Group 4—ultra-processed foods. The recipes of homemade dishes reported by subjects and the standard recipe database from the KNHANES (in the case of dishes consumed outside the home such as restaurants or cafeterias) were used to disaggregate the underlying ingredients of mixed dishes and classify each ingredient into NOVA food groups. This categorization was independently performed by a team of three researchers and the items were reclassified with discussions for nonconforming ones. Examples of each NOVA food group classified in this study are presented in Table 1.

### 2.4. Covariates

Data of sociodemographic variables (age, sex, household income, education level, marital status, and one-person household) and health-related lifestyle variables (smoking, alcohol consumption, and physical activity) were collected using face to face interviews and self-administered questionnaires, respectively [36,41]. Household income was calculated as the total household income divided by the root of the number of household members and divided into “lowest”, “lower middle”, “upper middle”, and “highest.” Smoking status was classified as “smoker” when the individual had smoked more than 5 packs of cigarettes (=100 cigarettes) in their lifetime and were currently smoking. Alcohol consumption was categorized as “alcohol drinker” if the individual had consumed alcohol more than once a month. Physical activity was regarded as “yes” in the case of performing exercise for more than 2 h 30 min of moderate-intensity physical activity or more than 1 h 15 min of high-intensity physical activity per week.

### 2.5. Statistical Analysis

Subjects were divided into quartiles according to relative energy intake (% of total energy intake) from ultra-processed foods (using sex-specific cut-offs). We used the relative energy intake from ultra-processed foods because total energy intake may be associated with ultra-processed food intake. The relative energy intake from ultra-processed foods was calculated as the daily energy intake from ultra-processed foods divided by total daily energy intake from all foods and multiplied by 100.

The categorical variables and continuous variables for general characteristics of subjects were calculated using PROC SURVEYFREQ and PROC SURVEYREG, respectively. Nutrient intake and the prevalence of nutrient inadequacy according to quartiles of relative energy intake from ultra-processed foods were compared using PROC SURVEYREG and PROC SURVEYFREQ procedures, respectively. Nutrient inadequacy was evaluated using the recommendation for the prevention of chronic diseases by the WHO [42,43,44]. Adjusted regression coefficients and 95% confidence intervals (CIs) using PROC SURVEYREG were calculated to evaluate the association between relative energy intake from ultra-processed foods and BMI and WC, respectively. We performed multivariable logistic regression analyses using PROC SURVEYLOGISTIC to estimate the odds ratios (ORs) and 95% CIs for the risk of obesity and abdominal obesity using BMI and WC after adjusting for covariates. We also conducted a stratified analysis by sex. *p* for trend across quartiles of relative energy intake from ultra-processed foods was calculated using the median value of each quartile as a continuous variable. All statistical analyses were performed using SAS, version 9.4 (SAS Institute Inc., Cary, NC, USA). We applied the primary sample units, stratification, and sampling weight in the analysis to consider the complex sampling design of KNHANES. *p* < 0.05 was considered to indicate statistical significance.

## 3. Results

### 3.1. General Characteristics of Subjects

Of all participants, 3219 were men (mean age 41.7 ± 0.3 years) and 4145 were women (mean age 42.8 ± 0.3 years). Mean daily energy intake was 2102.3 kcal; 57.5% of this energy was obtained from unprocessed or minimally processed foods, 3.8% from processed culinary ingredients, 11.9% from processed foods, and 26.8% from ultra-processed foods. The proportion of ultra-processed foods that contributed to the total energy consumption was higher among men than among women (28.4 vs. 25.1%, *p* < 0.0001).

Table 1 presents the distribution of total daily energy intake according to the NOVA food groups and subgroups in this study population. The main food groups contributing to total energy consumption were cereal grains and flours (32.0%), followed by meat and poultry (8.7%), fruits and vegetables (6.9%), and noodles and other grain products (5.8%). Among ultra-processed foods, beverages such as carbonated drinks, fruit drinks, and coffee (4.3%); industrialized bread, cakes, and bakery products (3.6%); sauce, dressing, and condiments (3.5%); and instant noodles (3.5%) were the highest contributors to total energy consumption. The proportion of energy intake from beverages (4.8 vs. 3.9%, *p* < 0.0001), distilled alcohol (4.8 vs. 1.2%, *p* < 0.0001), and instant noodles (3.9 vs. 3.0%, *p =* 0.0003) among men was higher than that among women, while the proportion of energy intake from industrialized bread and bakery products (3.0 vs. 4.3%, *p* < 0.0001), milk-based products (1.4 vs. 2.0%, *p* < 0.0001), snacks (1.7 vs. 2.1%, *p* = 0.0149), and confectionary (0.6 vs. 0.8%, *p =* 0.0182) was lower among men than among women.

We performed additional analysis to find if there is a difference in ultra-processed food consumption on weekdays and weekend days. The relative energy intake from ultra-processed foods on weekends was higher than that on weekdays among women (men: 29.4 vs. 27.8% *p* = 0.0565, women: 26.4 vs. 24.5%, *p* = 0.0138), but there was no significant difference after adjustment for age, household income, and education level (men: 29.4 vs. 27.8% *p* = 0.0514, women: 25.9 vs. 24.6%, *p* = 0.0559).

The relative energy intake from ultra-processed foods according to the general characteristics of the participants is shown in Table 2. Both the crude and adjusted relative energy intake from ultra-processed foods were higher among men, younger participants, smokers, and those with higher levels of education.

### 3.2. Energy and Nutrient Intakes According to Relative Energy Intake from Ultra-Processed Foods

Energy and nutrient intakes of subjects according to quartiles of relative energy intake from ultra-processed foods by sex are shown in Table 3. After adjusting for sociodemographic factors (age, household income, and education level), as the relative energy intake from ultra-processed foods increased, the intake of total energy (*p-*trend < 0.0001 for men, *p-*trend = 0.0030 for women), total fats, saturated fats, and sugars significantly increased (*p-*trend < 0.0001 for all). In contrast, the contents of carbohydrate, protein, dietary fiber, and potassium significantly decreased (*p-*trend < 0.0001 for all) in both men and women as the relative energy intake from ultra-processed foods increased.

Table 3 presents the prevalence of nutrient inadequacy by sex that did not meet the recommendation for the prevention of chronic diseases by the WHO [42,43,44] across quartiles of relative energy intake from ultra-processed foods. Compared with participants in the first quartile, the prevalence of inadequate nutrient intake of total fats, saturated fats, dietary fiber, potassium, and sugars, except sodium, was higher in those of the fourth quartile in both men and women.

### 3.3. Association between Ultra-Processed Foods and Obesity

Table 4 shows the results of multiple linear regression analysis and multiple logistic regression analysis for the association between ultra-processed food intake and BMI, WC, and obesity risk assessed by BMI and WC. When compared with the participants in the lowest quartile of relative energy intake from ultra-processed foods, the regression coefficients for BMI and WC of those in the highest quartile were unexpectedly lower (data not shown). However, after adjusting for sociodemographic (age, sex, household income, education level, residential area, marital status, and one-person household) and lifestyle (smoking status, alcohol consumption, and physical activity) variables, there were no significant associations in total participants. To further evaluate the association between ultra-processed food consumption and obesity, we conducted a stratified analysis by sex. After adjusting for sociodemographic and lifestyle variables, women in the highest quartile of relative energy intake from ultra-processed foods had 0.61 kg/m^2^ higher BMI (95% CI 0.23–0.99, *p*-trend 0.0047), 1.34 cm higher WC (95% CI 0.35–2.34, *p*-trend 0.0146), 51% higher odds of being obese (BMI >25 kg/m^2^; OR 1.51, 95% CI 1.14–1.99, *p*-trend 0.0037), and 64% higher odds of abdominal obesity (OR 1.64, 95% CI 1.24–2.16, *p*-trend 0.0004) than those in the first quartile. However, no significant associations were observed among men.

As total energy intake may be a mediator or a confounder in the association between ultra-processed food consumption and obesity, we performed additional analysis by adjusting for total energy intake, and the results did not substantially change (data not shown).

## 4. Discussion

In this nationally representative cross-sectional study, we found that Korean adults consumed 26.8% of energy from ultra-processed foods. As the consumption of ultra-processed foods increased, the dietary content of total fats, saturated fats, and sugars increased significantly, while the dietary content of protein, fiber, and potassium decreased. We also observed a positive association between the consumption of ultra-processed foods and obesity after adjusting for potential confounders in women, but not in men.

In our study, the relative energy intake from ultra-processed foods was similar to that found in Brazil (age ≥10 years, 20.4%) [8], Chile (age ≥ 2 years, 28.6%) [9], and Malaysia (age between 18 and 59 years, 24%) [45]. However, it was relatively low compared to that reported in other countries such as France (age between 18 and 86 years, 32%) [17], Japan (age 30–59 years, 38.2%) [46], Australia (age ≥ 2 years, 42.0%) [47], the USA (age ≥ 20 years, 55.5%) [15], and the UK (age ≥ 1.5 years, 56.8%) [7]. Even though Korea has shown dietary changes through processed food and food service industry development after rapid economic development and industrialization [30,31], Koreans have still maintained many of the aspects of the traditional diet based mainly on rice and vegetables [48,49]. These features might explain why the energy intake from ultra-processed foods in this study was lower than that observed in other countries.

We also found that the unadjusted relative energy intake from ultra-processed foods was higher among younger, single (never-married, separated, or divorced), and one-person households in the present study. In Japan, age and sex were not significantly associated with ultra-processed food consumption, but single (never married) and people who live alone consumed more ultra-processed foods [46]. In Korea, the number of one-person households has steadily increased, accounting for 30.2% of all households in 2019 [50]. A study conducted in Korea found that adults in one-person households in their 20s and 30s consumed more ready-to-eat foods than those in multi-person households [51]. Therefore, it is expected that the dietary contribution of ultra-processed foods will increase in Korea.

There was a significant positive association between the relative energy intake from ultra-processed foods and dietary intakes of total energy, total fats, saturated fats, and sugars in this study. In contrast, a strong inverse association was found between ultra-processed food consumption and the intake of carbohydrates, proteins, dietary fiber, and potassium in both men and women. Additionally, for total fats, saturated fats, dietary fiber, and potassium, the prevalence of inadequate nutrient intake based on WHO dietary recommendations for the prevention of chronic disease increased across the quartiles of relative energy intake from ultra-processed foods in both men and women. Although the prevalence of inadequate nutrient intake for total fats and saturated fats in the Korean population in this study was not relatively high, the prevalence of total fats and saturated fats in the highest quartile group was 2.8 and 4.3 times higher than that of the lowest quartile group among men and 3.3 and 4.3 times among women, respectively. Similar findings have been reported in previous studies conducted in Chile [9], the UK [7], and the USA [14]. In Chile, the prevalence of inadequate intake of free sugars, total fats, saturated fats, trans fats, potassium, and fiber (WHO recommendations) increased with the consumption of ultra-processed foods [9]. In the USA, as the consumption of ultra-processed foods increased, the content of protein, fiber, and micronutrients decreased, while the consumption of carbohydrate, added sugars, and saturated fats increased [14]. In the UK, the content of protein, fiber, and potassium decreased with the consumption of ultra-processed foods, while the content of carbohydrates, free sugars, total fats, saturated fats, and sodium increased [7].

In our study, there was no positive association between the dietary intake of sodium and ultra-processed food consumption, which has been observed in the UK [7] and Australia [47]. However, this result was similar to those conducted in the USA [14] and Portugal [52], which observed that the dietary intake of sodium decreased as ultra-processed food consumption increased. This may be because the main sources of sodium in those countries are different [7,14,52]. A study carried out in Korea found that the major dietary sodium sources were kimchi (27%) and added condiments (38%) such as salt, soy sauce, and soybean paste [53]. Most of these foods were not classified as ultra-processed food in our study.

The poor nutritional quality of ultra-processed foods may be associated with an increased risk of obesity because it is known that total fats, saturated fats, and free sugars have been associated with an increased risk of obesity, while dietary fiber has been associated with a reduced risk of obesity [42,54]. Several cross-sectional studies [22,23,24,25], cohort studies [26,27,28], and a randomized clinical trial [29] have shown an association between the consumption of ultra-processed foods and obesity. In Canada, participants in the highest quintile of ultra-processed food consumption had 32% higher odds of obesity (OR 1.32, 95% CI 1.05, 1.57) [23]. In a prospective study conducted in Spain, ultra-processed food consumption was associated with a higher risk of being overweight or obesity (HR 1.26, 95% CI 1.10, 1.45) [27]. A recent randomized controlled study conducted in the USA found that participants consumed more energy and gained body weight during the ultra-processed diet when they consumed ultra-processed diets or unprocessed diets were matched for presented calories, sugar, fat, fiber, and macronutrients for 2 weeks [29]. In our study, after adjusting for socioeconomic and lifestyle factors, we found that higher consumption of ultra-processed foods was significantly associated with higher BMI, WC, and odds of obesity (BMI ≥ 25 kg/m^2^) and abdominal obesity in women but not in men. This result suggests that there may be sex-based differences in the relationship between ultra-processed food intake and obesity in the Korean population.

The observed sex-based differences in the present study were similar to the results of other studies. In a cross-sectional study of Brazilians aged ≥10 years using data from 2008 to 2009, a significant association between ultra-processed food consumption and BMI and obesity was observed only in women [22]. Compared with the lowest intake group of ultra-processed foods, BMI was 1.13 kg/m^2^ higher (95% CI 0.38–1.87) and the odds of being obese were 1.96 in the highest intake group (95% CI 1.09–3.53) among women; however, BMI was only 0.32 higher (95% CI −0.36, 1.01) and odds of being obese were 1.06 (95% CI, 0.55–2.04) among men. In another cross-sectional study of adults aged 20–64 years from 2005 to 2014 in the USA, higher consumption of ultra-processed foods was associated with 2.37 kg/m^2^ higher BMI in women (95% CI 1.58–3.17) but only 0.79 kg/m^2^ in men (95% CI 0.18–1.39) [24].

Some researchers have explained the reasons for these sex-based differences; women tend to consume more sweetened products than men, and high glycemic index (GI) and glycemic load (GL) may have more adverse metabolic effects in women than in men [22,24,25]. In a study conducted in the USA, women consumed more high-sugar ultra-processed foods such as cake, ice cream, and desserts than men. The results showed that women had higher energy intake from carbohydrate and total sugars than men [24]. In a study carried out in Korea among adults aged 20–74 years, high dietary GI and GL showed a positive association with the prevalence of obesity in women but not in men [55]. These findings are consistent with our results. Indeed, the proportion of energy intake from bakery products such as bread and cakes (4.3 vs. 3.0%, *p* < 0.0001), milk-based products (2.0 vs. 1.4%, *p* < 0.0001), snacks (2.1 vs. 1.7%, *p* = 0.0149), and confectionary (0.8 vs. 0.6%, *p* = 0.0182) consumption was higher among women than among men in our study. Mean energy intakes from carbohydrates (men: 58.2%, women: 62.6%, *p* < 0.0001) and sugars (men: 11.4%, women: 14.0%, *p* < 0.0001) were higher among women than among men (results not shown). Further studies are required to determine other metabolic effects or unmeasured confounders to explain the sex differences.

In addition to the low nutritional quality of ultra-processed foods, several potential mechanisms have been suggested for the association between ultra-processed foods and obesity risk. Some researchers have proposed that food processing deconstructs the food matrix structure and thus may increase the eating rate, lower satiety, and higher glycemic response in experimental studies [29,56]. Others have suggested that food additives might be involved in obesity. Artificial sweeteners or food emulsifiers such as carboxymethylcellulose and Polysorbate-80 have been associated with the risk of developing glucose intolerance and obesity by altering the microbial composition [57,58]. Another mechanism may be related to compounds such as bisphenol or phthalates that are likely to leach into food products from the packaging of ultra-processed foods [59]. Bisphenol A or phthalate exposure has been associated with a higher risk of obesity and insulin resistance [60,61]. Lastly, the characteristics of ultra-processed foods such as hyper-palatability, convenience, large portion size, and persuasive marketing may also lead to overconsumption [22,62].

Some limitations of this study should be noted. First, considering its cross-sectional design, it is difficult to determine a causal association between ultra-processed food intake and obesity. Second, there is a possibility of NOVA misclassification error. Although we classified individual ingredients of mixed dishes into NOVA food groups using detailed information such as the recipe of homemade dishes or product brand from subjects and the standard recipe database from the KNHANES for more accurate estimation of the relative energy intake from ultra-processed foods, these data may be limited or may have been misclassified. Finally, dietary intakes were estimated using a one-day 24-h recall, which might not reflect a person’s usual diet. Despite these limitations, our study has several strengths. First, we used the most recent data from a nationally representative sample of Korea’s population. Second, to the best of our knowledge, this is the first study to evaluate the effects of consuming ultra-processed foods on obesity through the use of the NOVA classification system based on the nature, extent, and purpose of industrial food in the Korean population, while previous studies focused on nutrients of individual processed food or dietary pattern.

In conclusion, this study provides evidence that higher energy intake from ultra-processed foods was associated with poor dietary quality, which was characterized by a higher intake of total fats, saturated fats, and sugars and lower intake of dietary fiber and potassium. The high consumption of ultra-processed foods in women was associated with higher BMI, WC, and odds of obesity (BMI ≥ 25 kg/m^2^) and abdominal obesity. However, there was no such association in men. Further longitudinal studies or randomized controlled trials are needed to identify the mechanism of associations between ultra-processed food consumption and obesity in Korean adults.

## Figures and Tables

**Table 1:** Distribution (%) of the total daily energy intake (kcal) according to NOVA food groups among Korean adults ^(1)^.

	% of Total Daily Energy Intake						
	Total (*n* = 7364)		Men (*n* = 3219)		Women (*n* = 4145)		
	Mean	SE	Mean	SE	Mean	SE	*p* Value ^(2)^
**Unprocessed/minimally processed foods**	**57.49**	**0.31**	**56.10**	**0.43**	**59.05**	**0.38**	**<0.0001**
Cereal grains and flours	32.00	0.28	32.33	0.37	31.63	0.35	0.1243
Meat and poultry	8.66	0.15	9.47	0.22	7.74	0.19	<0.0001
Fruits and vegetables	6.92	0.11	5.51	0.11	8.50	0.15	<0.0001
Eggs	2.32	0.05	2.24	0.07	2.41	0.07	0.0525
Fish and seafood	2.16	0.05	2.15	0.07	2.18	0.07	0.7628
Milk and plain yoghurt	1.67	0.05	1.37	0.07	2.01	0.08	<0.0001
Potato and other roots	1.44	0.07	1.02	0.08	1.91	0.12	<0.0001
Legumes	0.78	0.03	0.70	0.03	0.87	0.03	<0.0001
Nuts and seeds	0.86	0.03	0.69	0.03	1.05	0.05	<0.0001
Others ^(a)^	0.68	0.01	0.62	0.02	0.75	0.02	<0.0001
**Processed culinary ingredients**	**3.83**	**0.05**	**3.77**	**0.07**	**3.89**	**0.07**	**0.2233**
Plant oils and animal fats	2.94	0.04	2.97	0.06	2.90	0.05	0.3254
Sugar, honey, and maple syrup	0.68	0.02	0.57	0.02	0.79	0.03	<0.0001
Traditional Korean fermented condiments ^(b)^	0.05	0.00	0.05	0.00	0.06	0.00	0.0604
Others ^(c)^	0.16	0.01	0.18	0.02	0.14	0.01	0.0285
**Processed foods**	**11.86**	**0.20**	**11.77**	**0.28**	**11.97**	**0.24**	**0.5525**
Noodles and other grain products ^(d)^	5.83	0.18	5.48	0.24	6.22	0.21	0.0100
Fruits, vegetables, and other preserved plant foods ^(e)^	2.11	0.03	2.04	0.04	2.18	0.05	0.0158
Fermented alcoholic drinks ^(f)^	1.93	0.08	2.27	0.12	1.54	0.10	<0.0001
Tofu	0.97	0.03	0.95	0.04	0.99	0.05	0.5071
Salted, smoked, or canned meat or fish	0.72	0.04	0.75	0.05	0.69	0.05	0.3525
Others ^(g)^	0.31	0.02	0.28	0.03	0.34	0.02	0.0988
**Ultra-processed foods**	**26.82**	**0.27**	**28.35**	**0.38**	**25.09**	**0.36**	**<0.0001**
Beverages	4.34	0.09	4.75	0.12	3.89	0.12	<0.0001
Instant coffee and coffee drinks	2.08	0.06	2.33	0.08	1.81	0.08	<0.0001
Carbonated drinks	1.12	0.05	1.34	0.07	0.87	0.06	<0.0001
Fruit drinks and other sugar added drinks	1.14	0.04	1.08	0.06	1.20	0.07	0.1504
Bread, cakes, and bakery products	3.62	0.12	3.00	0.16	4.32	0.17	<0.0001
Sauce, dressing, and condiments ^(h)^	3.53	0.06	3.49	0.08	3.58	0.08	0.3554
Instant noodles	3.46	0.14	3.89	0.21	2.98	0.16	0.0003
Distilled alcoholic drinks	3.08	0.14	4.75	0.23	1.21	0.12	<0.0001
Meat and seafood products ^(i)^	2.29	0.09	2.43	0.14	2.13	0.10	0.0672
Snacks	1.91	0.07	1.74	0.10	2.09	0.10	0.0149
Sweet snacks	1.34	0.06	1.19	0.08	1.49	0.09	0.0150
Salty snacks	0.57	0.04	0.55	0.06	0.60	0.05	0.5516
Milk-based drinks, processed cheese, and ice cream	1.67	0.06	1.35	0.08	2.04	0.10	<0.0001
Ready to eat, ready to heat products and other home meal replacements	1.11	0.06	1.11	0.09	1.11	0.08	0.9756
Fast foods (hamburger, pizza, and hotdog)	0.88	0.07	1.00	0.11	0.76	0.08	0.0650
Confectionary, jam, and ice pops	0.70	0.04	0.61	0.06	0.80	0.05	0.0182
Breakfast cereals	0.17	0.02	0.15	0.02	0.19	0.02	0.1935
Others ^(j)^	0.05	0.01	0.08	0.02	0.02	0.01	0.0194

^(1)^ Values are presented as mean ± standard error. ^(2)^ *p*-values were calculated using PROC SURVEYREG. ^(a)^ Includes herbs, dried spices, coffee and tea, edible insects, and yeast. ^(b)^ Includes soy sauce, soybean paste (doenjang in Korean), and red chili paste (gochujang in Korean). ^(c)^ Salt, vinegar, starch, coconut milk, gelatin, and baking powder. ^(d)^ Includes tteok (Korean rice cakes) and starch gel (e.g., muk in Korean). ^(e)^ Includes canned or bottled fruits or vegetables, Korean pickled vegetables (e.g., kimchi and jangajji in Korean), and salted seaweeds. ^(f)^ Includes wine, beer, and Korean rice wine (e.g., makgeolli and cheongju in Korean). ^(g)^ Includes cheese, salted or sugared nuts, and seeds. ^(h)^ Includes margarines, peanut butter, all syrups (excluding processed culinary ingredients), artificial sweeteners, and Korean fermented condiments’ added food additives. ^(i)^ Includes ham, sausages, chicken nuggets, fried chicken, and Japanese fish cakes. ^(j)^ Includes health and weight control food, and baby formula.

**Table 2:** Relative energy intake from ultra-processed foods according to the general characteristics of the participants among Korean adults ^(1)^.

			% of Total Daily Energy Intake from Ultra-Processed Foods					
	Distribution		Crude			Adjusted ^(2)^		
	*n*	(%)	Mean	SE	*p*-Value ^(3)^	Mean	SE	*p*-Value ^(3)^
**Sex**								
Men	3219	(52.9)	28.35	0.36	<0.0001	27.55	0.39	0.0165
Women	4145	(47.1)	25.09	0.38 **		26.19	0.38*	
**Age group (years)**								
19–29	1114	(21.1)	35.67	0.64	<0.0001	34.57	0.82	<0.0001
30–49	3301	(45.4)	27.73	0.38 **		27.53	0.42 **	
50–64	2949	(33.5)	20.01	0.35 **		20.64	0.41 **	
*p* for trend					<0.0001			<0.0001
**Household income level ^(4)^**								
Lowest	736	(9.5)	26.09	0.99	0.5724	26.22	0.94	0.4254
Lower middle	1792	(23.4)	27.36	0.56		27.58	0.53	
Upper middle	2284	(31.5)	26.91	0.42		26.64	0.40	
Highest	2543	(35.6)	26.51	0.46		26.80	0.46	
*p* for trend					0.7243			0.8007
**Education level ^(5)^**								
Middle school or lower	1268	(13.9)	20.26	0.60	<0.0001	24.98	0.66	0.0022
High school	2632	(39.1)	28.56	0.47 **		27.59	0.43 **	
College or higher	3136	(47.0)	27.46	0.37 **		26.81	0.37 *	
*p* for trend					<0.0001			0.2662
**Residential area**								
Urban	6121	(87.6)	27.11	0.30	0.0029	27.01	0.29	0.1138
Rural	1243	(12.4)	24.78	0.72 **		25.88	0.66	
**Marital status**								
Single/Separated/Divorced	2141	(34.5)	31.71	0.52	<0.0001	27.73	0.62	0.1169
Married	5222	(65.5)	24.24	0.30 **		26.43	0.37	
**Households**								
One-person household	646	(8.3)	29.42	0.95	0.0040	27.57	0.29	0.4439
Multi-person household	6718	(91.7)	26.58	0.28 **		26.81	0.91	
**Smoking ^(6)^**								
Non-smoker	5777	(75.7)	25.61	0.28	<0.0001	25.90	0.28	<0.0001
Smoker	1532	(24.3)	30.58	0.60 **		30.21	0.68 **	
**Alcohol drinker ^(7)^**								
Non-drinker	2893	(35.9)	24.69	0.41	<0.0001	26.25	0.42	0.0526
Drinker	4419	(64.1)	28.00	0.34 **		27.25	0.33	
**Physical activity ^(8)^**								
No	3919	(52.7)	26.54	0.38	0.1669	27.56	0.36	0.0091
Yes	3109	(47.3)	27.29	0.41		26.20	0.37 **	

^(1)^ Values are presented as number (weighted percent) or mean ± standard error. ^(2)^ Adjusted for all the other variables in the Table ^(3)^ *p-*values were calculated using PROC SURVEYREG. Bonferroni correction for multiple comparisons was conducted at alpha = 0.05 (first category as reference, *: *p* < 0.05, **: *p* < 0.01). ^(4)^ Household income was calculated as total household income divided by the root of the number of household members, and divided into “lowest”, “lower middle”, “upper middle”, and “highest.” The missing value is 9. ^(5)^ The missing value is 328. ^(6)^ Smokers: People who had smoked more than 5 packs of cigarettes (=100 cigarettes) in their lifetime and were currently smoking. The missing value is 55. ^(7)^ Alcohol drinker: People who drink more than once a month. The missing value is 52. ^(8)^ Physical activity: People who engaged in moderate-intensity physical activity for more than 2 h 30 min or high-intensity physical activity for more than 1 h 15 min per week. The missing value is 336.

**Table 3:** Nutrient intakes and prevalence of inadequate nutrient intake according to quartiles of relative energy intake from ultra-processed foods among Korean adults ^(1)^.

	Quartile of % Energy Intake from Ultra-Processed Foods																		
	Men (*n* = 3219)									Women (*n* = 4145)									
	Quartile 1 ^(2)^		Quartile 2		Quartile 3		Quartile 4		*p* for Trend ^(3)^	Quartile 1 ^(2)^			Quartile 2		Quartile 3		Quartile 4		*p* for Trend ^(3)^
	Mean	SE	Mean	SE	Mean	SE	Mean	SE		Mean		SE	Mean	SE	Mean	SE	Mean	SE	
Ultra-processed foods (% of total energy)	7.0	0.2	18.2	0.1	30.8	0.2	53.1	0.5	<0.0001	5.4		0.1	14.7	0.1	26.1	0.1	49.0	0.5	<0.0001
Total energy(kcal/d)	2160.9	31.8	2391.2	35.5	2497.4	35.5	2545.9	34.8	<0.0001	1708.4		26.5	1738.1	23.7	1773.7	21.7	1821.0	27.4	0.0030
Nutrient intake from (% of total energy)																			
Protein	15.8	0.2	15.4	0.2	14.3	0.2	12.9	0.2	<0.0001	14.9		0.2	15.0	0.2	14.6	0.1	13.3	0.2	<0.0001
Total fats	17.3	0.4	20.2	0.4	21.1	0.3	21.4	0.4	<0.0001	17.3		0.4	18.9	0.3	21.1	0.3	24.5	0.3	<0.0001
Saturated fats	5.1	0.1	6.5	0.1	6.9	0.1	7.6	0.1	<0.0001	5.1		0.1	5.9	0.1	7.0	0.1	8.7	0.1	<0.0001
Carbohydrate	64.7	0.5	61.0	0.5	57.3	0.5	51.6	0.6	<0.0001	66.7		0.5	64.4	0.4	61.7	0.4	58.4	0.5	<0.0001
Sugars	9.4	0.2	11.5	0.3	12.2	0.3	12.2	0.3	<0.0001	12.5		0.3	13.1	0.2	14.3	0.3	16.1	0.3	<0.0001
Dietary fiber (g/1000 kcal)	13.2	0.2	11.8	0.2	10.5	0.2	9.8	0.2	<0.0001	15.9		0.3	13.9	0.2	12.9	0.2	11.9	0.2	<0.0001
Sodium (mg/1000 kcal)	1850.5	35.8	1800.0	25.9	1816.3	30.2	1669.8	27.4	<0.0001	1729.1		36.3	1823.5	32.7	1791.7	33.2	1654.8	26.8	0.0088
Potassium (mg/1000 kcal)	1538.2	18.7	1433.3	17.3	1280.5	13.4	1104.4	13.7	<0.0001	1721.2		21.6	1621.6	17.7	1499.2	16.3	1303.7	15.9	<0.0001
**Prevalence of inadequate nutrient intake ^(^** **^4)^**																			
	***n***	**(%)**	***n***	**(%)**	***n***	**(%)**	***n***	**(%)**	***p*** **-value ^(^** **^5)^**	***n***	**(%)**		***n***	**(%)**	***n***	**(%)**	***n***	**(%)**	***p*** **-value ^(^** **^5)^**
Total fats (≥30% of energy)	48	(6.8)	91	(12.6)	106	(14.8)	144	(19.1)	<0.0001	79	(8.0)		89	(9.2)	133	(13.7)	279	(26.2)	<0.0001
Saturated fats (≥10% of energy)	42	(5.9)	91	(13.3)	107	(14.5)	187	(25.1)	<0.0001	79	(7.8)		96	(9.8)	155	(16.1)	341	(33.6)	<0.0001
Sugars (≥10% of energy)	297	(35.3)	456	(55.2)	448	(56.6)	446	(56.7)	<0.0001	574	(54.8)		675	(63.7)	729	(69.7)	782	(75.4)	<0.0001
Dietary fiber (≤12.5 g/1000 kcal)	358	(48.0)	470	(61.0)	587	(74.6)	631	(79.2)	<0.0001	316	(31.5)		431	(44.5)	553	(54.6)	727	(71.7)	<0.0001
Sodium (≥1000 mg/1000 kcal)	725	(90.0)	737	(91.8)	729	(91.2)	679	(84.4)	<0.0001	860	(82.7)		900	(86.6)	916	(87.9)	879	(84.3)	0.0170
Potassium (≤1755 mg/1000 kcal)	578	(74.7)	640	(80.4)	746	(92.8)	779	(97.0)	<0.0001	580	(55.2)		688	(66.0)	815	(80.3)	929	(88.9)	<0.0001

^(1)^ Values are presented as mean ± standard error or number (weighted percent). ^(2)^ Sex-specific quartiles of relative energy intake from ultra-processed foods cut-offs for quartiles were 12.5, 24.1, and 39.1 for men and 9.6, 19.7, and 33.5 for women, respectively. ^(3)^ *p* for trends calculated using the median value of each quartile as a continuous variable using PROC SURVEYREG. Adjusted for age, household income, and education level. ^(4)^ Indicators are recommended dietary nutrient goals for the prevention of chronic diseases specified by the WHO. ^(5)^ *p*-values were calculated using the Rao–Scott chi-square test for categorical variables.

**Table 4:** Odds ratio and confidence intervals/regression coefficient for obesity according to quartiles of relative energy intake from ultra-processed foods among Korean adults.

Quartile of % Energy Intake from Ultra-Processed Foods												
	Quartile 1 ^(1)^		Quartile 2			Quartile 3			Quartile 4			
Total (*n* = 7364)	β/OR	95% CI	β/OR	95% CI		β/OR	95% CI		β/OR	95% CI		*p* for Trend ^(2)^
BMI (kg/m^2^) (β)	0.0	Ref	0.03	−0.24	0.30	−0.06	−0.35	0.22	0.00	−0.28	0.29	0.9227
BMI ≥25 kg/m^2^ (OR)	1.0	Ref	0.92	0.77	1.10	0.92	0.76	1.10	0.96	0.80	1.16	0.8788
BMI ≥30 kg/m^2^ (OR)	1.0	Ref	0.94	0.66	1.35	0.90	0.62	1.32	0.88	0.61	1.26	0.4897
Waist circumference (cm) (β)	0.0	Ref	0.04	−0.69	0.77	−0.05	−0.80	0.70	0.04	−0.72	0.80	0.9534
Abdominal obesity (OR) ^(3)^	1.0	Ref	0.94	0.78	1.15	0.99	0.82	1.19	1.09	0.91	1.31	0.2154
***Men* (*n* = 3219)**												
BMI (kg/m^2^) (β)	0.0	Ref	−0.11	−0.51	0.29	−0.10	−0.52	0.31	−0.27	−0.68	0.15	0.2334
BMI ≥25 kg/m^2^ (OR)	1.0	Ref	0.84	0.66	1.06	0.91	0.71	1.16	0.81	0.64	1.03	0.1894
BMI ≥30 kg/m^2^ (OR)	1.0	Ref	0.75	0.44	1.28	0.98	0.60	1.59	0.69	0.42	1.15	0.2701
Waist circumference (cm) (β)	0.0	Ref	−0.29	−1.38	0.80	−0.03	−1.10	1.05	−0.45	−1.54	0.64	0.5091
Abdominal obesity (OR) ^(3)^	1.0	Ref	0.91	0.70	1.18	1.04	0.82	1.33	0.96	0.75	1.22	0.9842
***Women* (*n* = 4145)**												
BMI (kg/m^2^) (β)	0.0	Ref	0.32	−0.04	0.68	0.22	−0.14	0.59	0.61	0.23	0.99	0.0047
BMI ≥25 kg/m^2^ (OR)	1.0	Ref	1.14	0.87	1.48	1.06	0.80	1.40	1.51	1.14	1.99	0.0037
BMI ≥30 kg/m^2^ (OR)	1.0	Ref	1.32	0.80	2.17	0.81	0.46	1.45	1.42	0.84	2.40	0.3288
Waist circumference (cm) (β)	0.0	Ref	0.74	−0.22	1.69	0.55	−0.41	1.51	1.34	0.35	2.34	0.0146
Abdominal obesity (OR) ^(3)^	1.0	Ref	1.08	0.81	1.43	1.03	0.76	1.40	1.64	1.24	2.16	0.0004

Abbreviation: BMI, body mass index. ^(1)^ Sex-specific quartiles of relative energy intake from ultra-processed foods cut-offs for quartiles were 12.5, 24.1, and 39.1 for men and 9.6, 19.7, and 33.5 for women, respectively. ^(2)^ *p* for trend was calculated using the median of each quartile as a continuous variable through PROC SURVEYREG and PROC SURVEYLOGISTIC. Adjusted for age, sex, household income, education level, residential area, marital status, one-person household, smoking, alcohol consumption, and physical activity. ^(3)^ Abdominal obesity was defined as waist circumference ≥90 cm for men and ≥85 cm for women.

## Data Availability

Data were obtained from the Korean National Health and Nutrition Examination Survey (KNHANES) and are available from the KNHANES website (at http://knhanes.cdc.go.kr).
